# Editorial: Qualitative pain research: Capturing and integrating cultural, social and linguistic data

**DOI:** 10.3389/fpain.2022.1082044

**Published:** 2022-11-24

**Authors:** Najmeh Khalili-Mahani, Dominik Mischkowski, Richard B. Hovey

**Affiliations:** ^1^ McGill Centre for Integrative Neuroscience, Montreal Neurological Institute, Montreal, QC, Canada; ^2^ Culture and Mental Health Research Unit, Lady Davis Institute, Jewish General Hospital, Montreal, QC, Canada; ^3^ PERFORM Centre, Concordia University, Montreal, QC, Canada; ^4^ Department of Psychology, Ohio University, Athens, OH, United States; ^5^ Faculty of Dental Medicine and Oral Health Sciences, McGill University, Montreal, QC, Canada

**Keywords:** qualitative research and analysis, pain research, patient–centered care, cultural medicine, indigenous methodology, phenomenology, hermeneutics, metaphors of illness

**Editorial on the Research Topic** Qualitative pain research: Capturing and integrating cultural, social and linguistic data

In 2021, the International Association for the Study of Pain (IASP) added chronic pain to the WHO's International Classification of Disease (ICD-11) (1). Despite important progress in biomedical understandings of pain, major challenges remain to translate molecular and system medicine advances into clinical practice. According to the co-chairs of IASP's 2022 Global Year for Translating Knowledge to the Patient, such challenges include placebo effects, a persistent lack of molecular or clinical biomarkers of pain, and patient stratification for clinical trials (2). In addition, psychosocial and economic conditions interfere with access to medical pain management (2). Craig's Social Communication Model of Pain asserts that because humans must adapt to complex social environments, cognitive and social processes and contexts must be considered in understanding patients’ own perceptions and behaviors in presence of pain (3). Kirmayer's Cultural Somatization model explains that because pain experience is mediated, elaborated and communicated through cultural models, it needs to be studied more thoroughly with appropriate qualitative methodologies and culturally grounded approaches (4, 5).

To date, the impact of qualitative research in healthcare remains limited (6), though qualitative research can improve care (7) by enriching clinical encounters (8) and patient-engagement in research (9, 10). It can also influence policy-making (11). There are both epistemological challenges [e.g., a reductionist view on replicability (7) and “quality” (12)] and institutional biases [e.g., funding (13)] that prevent the implementation of extensive qualitative research into clinical practices.

To address this shortcoming, we focused on Cultural, Social and Linguistic aspects of qualitative pain research in this special issue, building upon our own perspectives as both qualitative and quantitative pain researchers [e.g., (14–16)]. The following themes emerged within this collection:

## Pain scales do not make sense for long term chronic pain treatment

Six articles in this Research Topic provide examples of how storytelling is essential to creating holistic patient-centered treatment programs.

Specifically, verbal and visual metaphors provide a strategy for pain communication. In *Lives Penciled In*, writing from a personal experience, Hovey et al. explain, “that health resists universal definition because it can only be interpreted, constituted, and reconstituted through specific professional, personal, promotional, educational, cultural, governmental, and communal lenses” and uses hermeneutics and the metaphor of “pencil” to draw the attention of healthcare providers to the fragility and brevity of healthcare encounters from the perspective of a cancer patient. Not only words, but drawings can help clinicians gain a more holistic view of how their patients progress over time. In *Portraying Improvement in the Management of Chronic Pain*, Nizza et al. examine the drawings of patients in a pain management program to illustrate their changing attitudes towards themselves and their pain over the course of six months.

Furthermore, two Indigenous studies in this collection illustrate that metaphors are culturally relevant. In *Clinical Strategies to Develop Connections, Promote Health and Address Pain From the Perspectives of Indigenous Youth, Elders, and Clinicians*, VanEvery et al. noted both intergenerational and intercultural differences in metaphors of pain, requiring sensitivity to not only historical and cultural backgrounds, but also literacy, linguistic abilities and inter- and intra-community connections when conducting research about the pain experience. In *A Window into Pain: American Indian Cancer Survivors' Drawings*, Hodge et al. assembled in 13 focus groups more than 130 adult American Indian cancer survivors and their caregivers living in urban and reservation communities in the Southwest USA. This research revealed the limitations of various standard pain scales as opposed to personal drawings of pain, suggesting that a mechanistic view of pain and cultural incongruity among patients and caregivers can lead to neglect and undertreatment of serious pain conditions.

Finally, storytelling constitutes an effective and preferred way of making sense of pain and sharing the pain experience with others. In *Beyond Pain Scales*, Miglio and Stanier concur that metaphors (verbal or visual) create a cognitive and emotional space for imagination and overcoming the limits of language, thus increasing collective understandings of chronic pain, and note that sharing the experience of pain through storytelling creates a sense of belonging and community. Furthermore, in *Toward a Digital Citizen Lab for Capturing Data About Alternative Ways of Self-Managing Chronic Pain*, Khalili-Mahani et al. report that the idea of creating a narrative-based participatory action is conceptually acceptable and desired by chronic pain patients. Indeed, Holowka's social-media research in *Mediating Pain: Navigating Endometriosis on Social Media* confirms the importance of collective sense-making and community-building in online support spaces, achieved through storytelling in social media by helping women to make their experience of endometriosis visible and describing its pain manifestations in their own terms.

## Qualitative research needs to be sensitive to cultural differences

In addition to the two Indigenous studies listed above, two other mixed-methods studies in this collection emphasize the cultural underpinnings of qualitative research. In *Development of a Mixed Hypnosis and Music Intervention Program for the Management of Pain, Anxiety, and Wellbeing in End-of-Life Palliative Care*, Bissonnette et al. report concerns expressed by clinicians that metaphors or music might increase the emotional burden and increase a patient's level of distress. Examining the *Experiences of Community-Dwelling Older Adults with Chronic Low Back Pain in Hong Kong and Switzerland*, Schoeb et al. demonstrate that in addition to cross-country differences in availability and accessibility of healthcare, significant cultural differences in self-perceived roles within a family contribute to variations in seeking support and coping with chronic lower back pain. Thus, in an epistemological culture that is heavily dominated by quantitative research practices, to undertake qualitative studies may become challenging due to lack of familiarity with its paradigms. For example, despite showing interest in a participatory digital citizen lab for qualitative research (Khalili-Mahani et al.), and finding data retrieved from self-expression to be just as important as pain tracking, the concept of storytelling for reporting their pain remained foreign to respondents who lived with chronic pain.

## Holistic and critical research frameworks enculture qualitative research

Several studies in this collection demonstrate that understanding cultural contexts is essential to providing good care and offer various theoretical and conceptual frameworks to capture the cultural dimensions of communicating pain.

In this collection, two holistic frameworks from Indigenous research methodologies were proposed. The LISTEN (*Language, Individual, Share, Teachable moments, Engage, and Navigate)* framework was guided by two pillars in Indigenous Epistemologies: ‘Two-Eyed Seeing’ and ‘The Medicine Wheel’ (VanEvery et al.). Both approaches provide the context for creating more culturally-grounded and safer practices for clinicians with the goal to manage pain and hurt more holistically. Consistent with this view, the Humanistic Nursing Theory acknowledges that “each person faces the end of life in a way that represents his or her unique life experience in the world”, in order to provide culturally-sensitive care to cancer survivors (17).

The Affect Theory and Feminist Social Media Studies offer a critical framework to expose the legacy of discriminatory and gendered dismissal of pain in minorities and women [(18) in Holowka]. In this context, Miglio and Stanier suggest critical phenomenology as a framework to capture the socio-political dimensions which frame painful experiences, their expression, their lived significance, and their treatment. Furthermore, Khalili-Mahani et al. use the Transactional Theory of Stress and Coping to frame individual variations in adaptation to chronic pain. Nizza et al. offer that interpretative longitudinal phenomenological methods could map the dynamic trajectory of an individual's experience of pain treatment.

## Conclusion

In conclusion, this Research Topic provides a glimpse into how cultural, linguistic, social and personal factors contribute to the authentic communication of the pain experience. The research studies featured in this collection offer different holistic and critical frameworks for qualitative research that can address current challenges in translational research through a community-based, patient-centered and encultured care plan.
